# Crystallization of amorphous silicon thin films deposited by PECVD on nickel-metalized porous silicon

**DOI:** 10.1186/1556-276X-7-464

**Published:** 2012-08-17

**Authors:** Sonia Ben Slama, Messaoud Hajji, Hatem Ezzaouia

**Affiliations:** 1 Laboratoire de Photovoltaïque, Centre des Recherches et des Technologies de l’Energie (CRTEn), Technopôle de Borj-Cédria, BP 95, Hammam-Lif, 2050, Tunisia; 2 Institut Supérieur d’Electronique et de Communication de Sfax (ISECS), Route Menzel Chaker Km 0.5, BP 868, Sfax, 3018, Tunisia

## Abstract

Porous silicon layers were elaborated by electrochemical etching of heavily doped p-type silicon substrates. Metallization of porous silicon was carried out by immersion of substrates in diluted aqueous solution of nickel. Amorphous silicon thin films were deposited by plasma-enhanced chemical vapor deposition on metalized porous layers. Deposited amorphous thin films were crystallized under vacuum at 750°C. Obtained results from structural, optical, and electrical characterizations show that thermal annealing of amorphous silicon deposited on Ni-metalized porous silicon leads to an enhancement in the crystalline quality and physical properties of the silicon thin films. The improvement in the quality of the film is due to the crystallization of the amorphous film during annealing. This simple and easy method can be used to produce silicon thin films with high quality suitable for thin film solar cell applications.

## Background

The use of porous silicon as intermediate layer for silicon thin film solar cells has attracted many research groups in the last decade [1-3]. A porous silicon (PS) layer with double porosity is generally used. The upper PS layer with low porosity serves as a seeding layer for epitaxial growth. The second layer with high porosity is used to (1) prevent pore filling during silicon deposition, (2) act as a gettering barrier, and (3) make easy the separation process of the device. The use of porous silicon as a substrate for silicon thin film deposition has many advantages if compared with foreigner substrates such as glass or ceramics: porous silicon can act as a barrier that prevents the diffusion of impurities from the substrate to the film. It can also support high temperatures required for solar cell processing (doping, metallization, etc.). The elaborated solar cell can be transferred to a low cost substrate (glass, ceramic, plastic, etc.). On the other hand, the silicon substrate can be used several times after the transfer of the solar cell.

The major problem for such structure, if the porous layer is not removed, is its high series resistance. One possible way to overcome this problem is the metallization of the intermediate PS layer. There are many techniques for the metallization of porous silicon such as vapor deposition, sputtering, and electrodeposition. Among these techniques, immersion plating is the simplest and the most economical method [4,5]. In this work, we present a study on the metallization of porous silicon by immersion in Ni solution and the use of the obtained material as a substrate for the deposition and crystallization of amorphous silicon thin films intended for thin film solar cell application. The presence of Ni on the porous structure walls will play a crucial role both in the crystallization process and the back metallic contact quality that will be realized after the transfer process of the thin silicon solar cells.

## Methods

Porous silicon was elaborated by electrochemical anodization of heavily boron-doped p-type silicon substrate in HF solution. Two current densities were successively used. A low density of 5 mA/cm^2^ was used to form an upper thin porous layer with low porosity of about 30% and a higher density of 50 mA/cm^2^ to form a deeper layer with high porosity of about 60%. Metallization of the porous layer was carried out by simple immersion in diluted nickel solution. Amorphous silicon (a-Si) thin films were deposited on metalized porous silicon substrates using plasma-enhanced chemical vapor deposition (PECVD) by decomposition of SiH_4_ (3 sccm) electronic grade mixed with H_2_ (150 sccm) at a reaction pressure of 133.3 Pa. The substrate temperature and the RF power were fixed to 300°C and 60 W, respectively. Obtained films were thermally annealed under vacuum at 750°C for 2 h. Structural and optical properties of crystallized silicon thin films were systematically analyzed by atomic force microscopy (AFM), X-ray diffraction (XRD), Raman spectroscopy, and UV–vis spectrophotometer.

## Results and discussion

**Figure 1:**
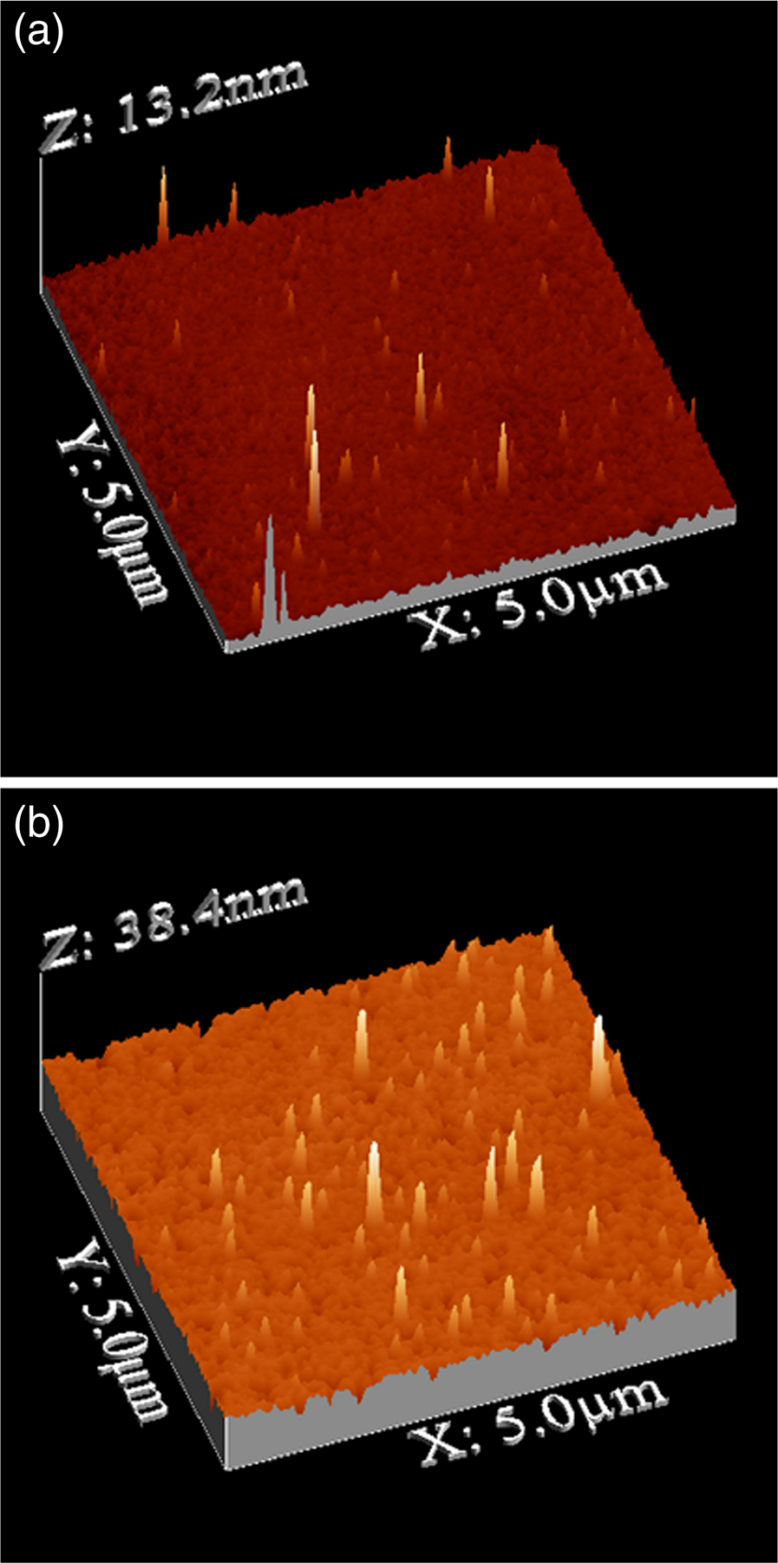
AFM images of as-prepared porous silicon before (a) and after (b) immersion in nickel solution.

In Figure 1, we present AFM images of porous silicon layers before and after immersion for 10 min in the nickel solution. The images show a significant change in the surface morphology of porous silicon after immersion. This change is attributed to Ni deposition on the porous silicon layer during the immersion in Ni solution.

**Figure 2:**
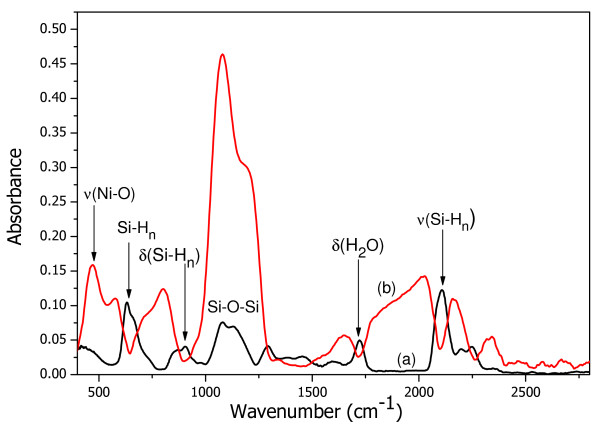
FTIR spectra of metalized porous silicon before (curve a) and after (curve b) oxidation.

Fourier transform infrared spectroscopy (FTIR) measurements are carried out to study the change in the chemical composition of porous silicon after immersion in Ni solution and to show in which form Ni is deposited on the porous surface. For these measurements, the PS layer was elaborated the on p-type silicon. As shown in Figure 2 (curve a), the present bands in porous silicon after immersion in Ni solution are the same as those observed in pure porous silicon. Observed FTIR bands are located at around 2,050 to 2,200 cm^−1^ (stretching modes), 800 to 1,000 cm^−1^ (bending modes), and 600 to 750 cm^−1^ (wagging modes) associated with Si-H_*n*_ (*n* ≥1) bondings, and finally, the band at 1,000 to 1,300 cm^−1^ corresponds to the stretching modes of the Si-O-Si bonds in the SiO_*x*_. An absorption band at approximately 1,720 cm^−1^ is produced by the bending mode of H_2_O. Obtained results do not give any indication on the deposition of nickel on the porous surface since at this stage, no band corresponding to nickel bonds was observed. Another way to identify the presence of Ni on PS is through the oxidation of the obtained material. This step was carried out just to prove the presence of Ni, and it will not be used for subsequent applications. After oxidation, there appears a new band centered at 470 cm^−1^ which is generally attributed to Ni-O bonds. The formation of NiO after oxidation is an indication that nickel is deposited on porous silicon in its metallic form and not in the form of its oxide.

**Figure 3:**
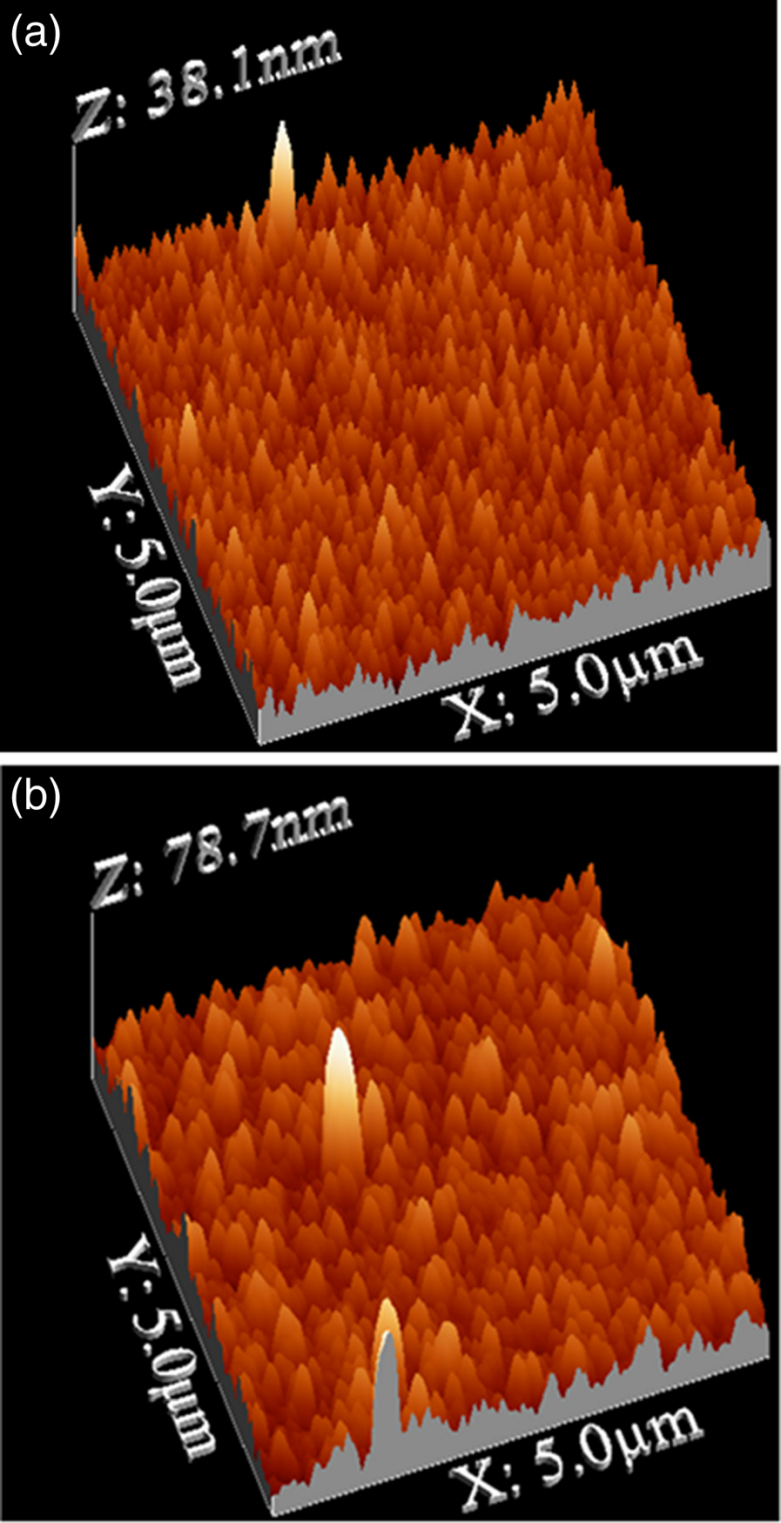
AFM images of a-Si/PS before (a) and after (b) crystallization.

Figure 3a shows an AFM image of an a-Si thin film prepared by PECVD on porous substrate. The image in Figure 3b was taken for the same structure after thermal annealing at 750°C for 2 h. These images show that after annealing, the sample is composed of large crystallites as compared with the untreated one. This behavior is due to the coalescence between grains during the crystallization process.

**Figure 4:**
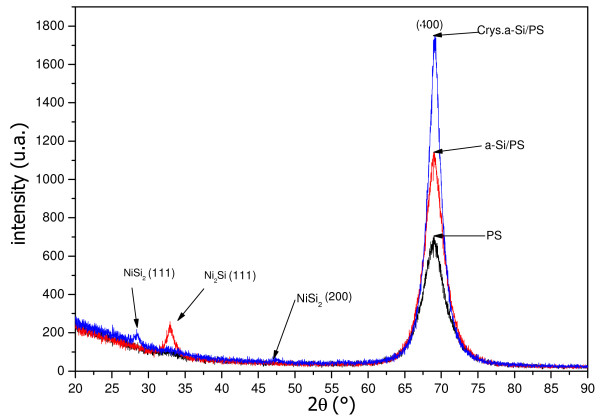
XRD patterns of PS, a-Si/PS, and film obtained after annealing of the structure a-Si/PS.

XRD was used to probe the crystallinity of the films after annealing. Figure 4 shows XRD patterns of the PS, a-Si on porous silicon substrate (a-Si/PS), and the films obtained after annealing of the a-Si/PS structure. The PS spectrum presents a single peak centered at about 2*θ* = 69.01° corresponding to the (004) plane. The spectrum of the a-Si shows two peaks: the first at 2*θ* = 69.01° from Si (400) planes, and the second weak peak at 2*θ* = 32.94° from Ni_2_Si (111) planes. The appearance of the Ni_2_Si phase is due to the reaction between the deposited a-Si and Ni during the deposition step. The peak in the a-Si spectrum is more intense than in the PS spectrum, indicating that the deposited a-Si film contains a crystalline phase. After thermal annealing, the peak shifts to 2*θ* = 69.12°, and its intensity increases, indicating an improvement in the crystalline quality of the film.

**Figure 5:**
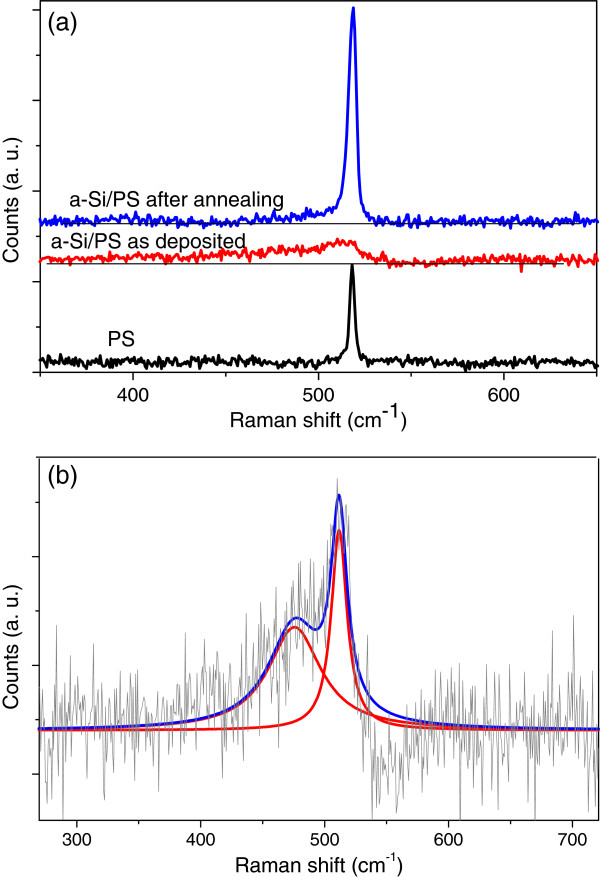
**Raman scattering spectra of a PS layer and a-Si/PS structure.** Before and after annealing (**a**) and the deconvoluted spectrum of a-Si/PS structure (**b**).

Raman spectroscopy was used to quantitatively characterize the crystallinity degree of the porous substrate and the a-Si thin films before and after annealing. The obtained Raman spectra are shown in Figure 5a. For the porous layer, there is only a sharp peak located around 518 cm^−1^ which is lower compared with the single crystalline silicon peak (520 cm^−1^). The shift to lower frequencies is attributed to the tensile stress in the porous layer [6]. The as-deposited a-Si layer presents a broad band that can be deconvoluted into two peaks (Figure 5b). The broad peak centered at about 476 cm^−1^ corresponds to the amorphous silicon [7,8], and the narrowest one located at 511 cm^−1^ is generally ascribed to transverse optic mode of the phonon in nc-Si [8-12]. This result suggests that the a-Si layer contains a crystalline phase with crystallites in the nanometer scale. For this sample, no peak was observed at 518 cm^−1^, indicating that the detected signal is only due to the response of the amorphous layer, and it does not contain any contribution from the porous substrate. After annealing at 750°C, the peak at 476 cm^−1^which corresponds to the amorphous phase disappears, and the peak attributed to crystalline phase shifts from 511 to 518.8 cm^−1^. This shift to the higher frequencies of the crystalline component is accompanied by a large reduction of the full width at half maximum from 15.5 to 5.3 cm^−1^. These results indicate that the crystallinity degree of silicon films is well improved after thermal annealing of the a-Si layer on Ni-metalized porous silicon and after a reduction in stress.

**Figure 6:**
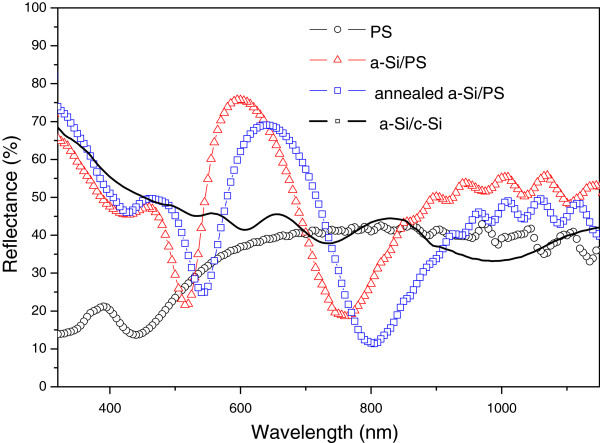
Optical reflectance spectra of PS, a-Si/PS, a-Si/c-Si, and film obtained after annealing of a-Si/PS structure.

Figure 6 presents the total reflectance spectra of PS double layer (PS), the as-deposited a-Si/PS, the as-deposited a-Si on polished monocrystalline silicon substrate (a-Si/c-Si), and a-Si/PS after annealing. The spectrum corresponding to the annealed sample shows an increase in the reflectance in the UV region which is another confirmation of the crystallization of the film. The film thickness was evaluated from the fitting of the a-Si/c-Si spectrum and was estimated to be 318 nm.

Table 1 summarizes the results obtained from four point probe measurements for a-Si/PS before and after thermal annealing. Results show a strong decrease in sheet resistance and the electrical resistivity of the layer after annealing. This decrease in the resistivity is due to an improvement in the crystalline quality of the layer.

**Table 1:** Sheet resistance and resistivity of a-Si thin film before and after annealing

**Sample**	**Sheet resistance*R*s (kΩ)**	**Resistivity ρ (Ω cm)**
a-Si/PS	193.36	6.148
Annealed a-Si/PS	1.515	0.048

Obtained results from structural, optical, and electrical characterizations show that thermal annealing of amorphous silicon deposited on Ni metalized porous silicon leads to an enhancement in the crystalline quality and physical properties of the silicon thin films. The improvement in the quality of the film is due to the crystallization of the amorphous film during annealing. In this case, crystallization is controlled by the presence of silicon crystallites of the porous layer (solid phase crystallization) that act as nucleation centers and the diffusion of nickel in silicon film (metal-induced crystallization) to form nickel silicides (Ni_2_Si, NiSi, NiSi_2_, etc.). The presence of these silicides, having a generally low resistivity, at the interface Si/PS is very important for the realization of metallic contacts with low resistance. This simple and easy method can be used to produce silicon thin films with high quality suitable for thin film solar cell applications.

## Conclusions

Deposition and crystallization of amorphous silicon thin films on Ni metalized porous silicon layer with double porosity were studied. Results show that amorphous silicon thin films were fully crystallized and preferentially <400 > oriented. This method seems to be very interesting for the production of high quality silicon thin films that can be used for the production of efficient and freestanding thin solar cells.

## Competing interests

The authors declare that they have no competing interests.

## Authors’ contributions

SBS carried out all the experiments of elaboration and characterizations and participated in the interpretation of the results. MH co-supervised the work, participated in the concept of the study, and wrote the manuscript. HE supervised the work and revised the manuscript. All authors read and approved the final manuscript.
